# How I manage anemia related to myelofibrosis and its treatment regimens

**DOI:** 10.1007/s00277-023-05126-4

**Published:** 2023-02-14

**Authors:** Srdan Verstovsek

**Affiliations:** Department of Leukemia, The University of Texas MD Anderson Cancer Center, Houston, TX 77030 USA

**Keywords:** Myelofibrosis, Anemia, Ruxolitinib, Myeloproliferative neoplasm, Janus kinase

## Abstract

Myelofibrosis (MF) is a myeloproliferative neoplasm characterized by mutations (most frequently in *JAK2*, *CALR*, or *MPL*), burdensome symptoms, splenomegaly, cytopenia, and shortened life expectancy. In addition to other clinical manifestations, patients with MF often develop anemia, which can either be directly related to MF pathogenesis or a result of MF treatment with Janus kinase (JAK) inhibitors, such as ruxolitinib and fedratinib. Although symptoms and clinical manifestations can be similar between the 2 anemia types, only MF-related anemia is prognostic of reduced survival. In this review, I detail treatment and patient management approaches for both types of anemia presentations and provide recommendations for the treatment of MF in the presence of anemia.

## Introduction

Myelofibrosis (MF) is a myeloproliferative neoplasm (MPN) characterized by abnormal megakaryocyte proliferation, along with reticulin or collagen fibrosis [1]. Nearly all patients (≈90%) have activating mutations in either *JAK2*, *CALR*, or *MPL*, which cause abnormal signaling that promotes cell proliferation and survival, as well as activation of several inflammation pathways [2–6]. MF clinical manifestations typically include anemia, thrombocytopenia, splenomegaly, and hepatomegaly that when combined can lead to burdensome symptoms such as fatigue, abdominal discomfort, night sweats, bone pain, and pruritus that impact patients’ quality of life [7, 8]. In addition to these burdensome signs and symptoms, patients with MF have increased risk of thrombosis and increased risk of progression to acute leukemia, which both also contribute to reduced survival compared with healthy controls [9, 10].

Anemia, at times reaching severe levels (< 8 g/dL), can be present at MF diagnosis and worsen over time as disease progresses (MF-related anemia), or it can manifest as a result of MF treatment with Janus kinase (JAK) inhibitors (treatment-related anemia) [11–15]. Although symptoms and clinical manifestations can be similar, only MF-related anemia is prognostic of reduced survival [12–15]. This review provides guidance for managing patients with either type of anemia presentation.

## Sample patient—part 1

A 68-year-old female patient presented with shortness of breath. During a physical examination, she was found to have an enlarged spleen of 7 cm below the costal margin and no other significant findings. She also reported fatigue, significant night sweating, and some weight loss. Laboratory results indicated hemoglobin (Hb) of 9.7 g/dL, a white blood cell (WBC) count of 22 × 10^9^/L with 2% blasts, and a platelet count of 122 × 10^9^/L. Lactate dehydrogenase and erythropoietin (EPO) were both elevated (1780 U/L and 35 mU/mL, respectively). Furthermore, a bone marrow biopsy was compatible with MF.

### General treatment of MF

**Fig. 1:**
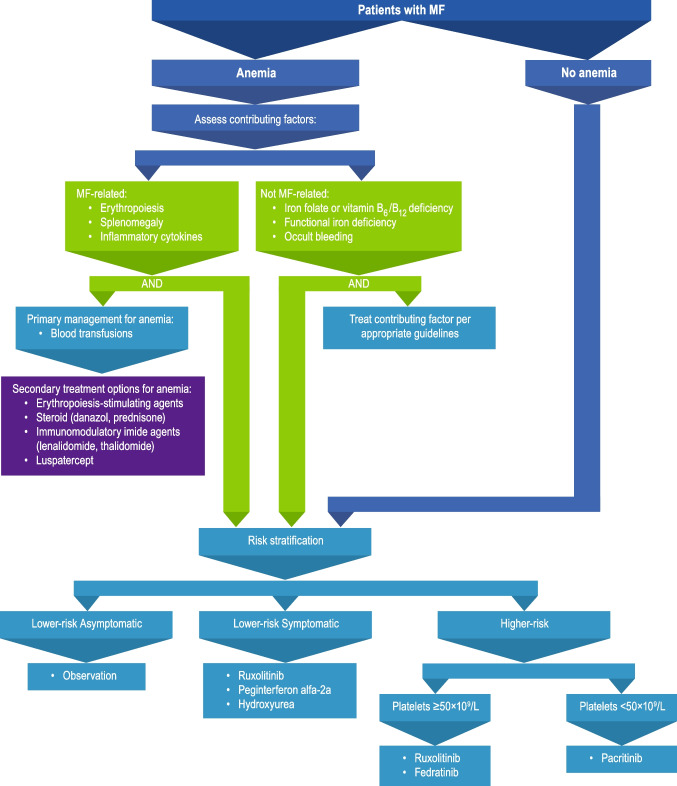
Treatment flow chart for patients with MF-associated anemia. Hematopoietic stem cell transplant should also be considered for eligible patients with high-risk disease. MF, myelofibrosis

In my practice, we would first determine a prognosis of a patient by risk stratification (Fig. 1). There are several prognostic scoring systems in use, among which the Mutation and Karotype-Enhanced IPSS (MIPSS-70 + VERSION 2.0) is probably the most comprehensive, with additional options including Dynamic International Prognostic Scoring System (DIPSS)-Plus if molecular testing is not available, DIPSS if karyotyping is not available, and Myelofibrosis Secondary to PV and ET-Prognostic Model (MYSEC-PM) for secondary MF. We consider a patient to be at higher risk if their risk score corresponds to high, intermediate-2, or a score in the higher intermediate range, consistent with the National Comprehensive Cancer Network guidelines [16]; such patients are typically referred to a stem cell transplant specialist for consideration of transplant procedure.

For patients with symptomatic disease, either lower- or higher-risk MF, typical treatment choice is the oral selective JAK1/JAK2 inhibitor ruxolitinib [16]. Ruxolitinib was the first JAK inhibitor approved for the treatment of MF by the US Food and Drug Administration in 2011 [17], and treatment prolongs survival, reduces symptom burden, and reduces spleen volume, as demonstrated by multiple clinical trials [18–20]. Risk of death, as assessed by the prognostic scoring systems mentioned above, does not guide our decision on prescribing medications to control symptoms. Our alternative choice to ruxolitinib is the JAK2 inhibitor fedratinib, approved in the US for patients with intermediate-2 or high-risk MF, which we typically use in the second-line setting [16, 21]. For cytoreduction of high WBC or platelet counts, which are sometimes seen in patients with lower-risk, early, or prefibrotic MF, peginterferon alfa-2a or hydroxyurea are used [16]. For higher-risk patients with MF with severe thrombocytopenia (platelets < 50 × 10^9^/L), pacritinib, an oral selective JAK2 and interleukin-1 receptor-associated kinase (IRAK1) inhibitor, is my preferred first-line treatment option [16, 22, 23]. Although both fedratinib and pacritinib provide clinical benefits, longer follow-up studies are required to determine if either provides an overall survival benefit as seen with ruxolitinib [23, 24].

### Treatment options for anemia resulting from MF

Mild to moderate anemia is often present at MF diagnosis and can worsen with disease progression. Importantly, unlike JAK inhibitor treatment–related anemia discussed later in this review, MF-related anemia is associated with reduced overall survival, so proper management is essential [14]. An overview of treatment recommendations for patients with MF-related anemia in my practice is presented in Fig. 1 [16]. Patients should first be evaluated for contributing factors, including MF-related factors and exacerbating causes not directly related to MF. MF-related causes include reduced erythropoiesis, splenomegaly, and inflammatory cytokines [12, 25]. Additionally, vitamin B_6_, an essential element of heme synthesis, may be deficient in patients with primary or secondary MF, leading to anemia [26]. Functional iron deficiency due to inflammation is also frequently observed in patients with MF [27, 28], in which pro-inflammatory cytokine signaling upregulates hepcidin that in turn promotes storage of iron and ultimately iron-restricted anemia [29]. Functional iron deficiency is identifiable by low transferrin saturation despite normal ferritin levels [27, 28], and these patients should be treated with intravenous iron [29]. In addition, new targeted therapies are in development to modulate hepcidin signaling, including the JAK1/JAK2 and type 1 kinase activin A receptor or activin receptor-like kinase-2 (ACVR1/ALK2) inhibitor momelotinib (discussed in greater detail in the “Treatment options for anemia resulting from JAK inhibition” section) [30, 31] and the ACVR1/ALK2 inhibitor INCB000928 [32]. Although seemingly very rare, cases of patients developing primary MF and autoimmune hemolytic anemia have been reported [33]. Exacerbating causes not directly related to MF include underlying occult or gastrointestinal bleeding and deficiencies in iron folate and vitamin B_12_, which can lead to megaloblastic anemia [12, 25, 34, 35]. Deficiencies in iron folate and vitamin B_12_ are not uncommon in elderly patient populations [36] such as the MF population and are reversible via dietary or vitamin supplementation [35]. For patients with contributing factors not related to MF, the underlying cause should be treated per appropriate guidelines, and patients should be treated normally for MF, regardless of anemia presence [16, 35].

Management of patients with MF-related anemia begins with blood transfusions, with subsequent evaluation for additional anemia treatments [16]. For patients with serum EPO < 500 mU/mL, erythropoiesis-stimulating agents (ESAs) are a viable option that offers clinical benefits [16]. Up to half of the patients in this population may achieve an anemia response with ESAs, and dose escalation should be considered to achieve full benefit [37]. Importantly, ESAs can be safely added to ruxolitinib to effectively improve anemia in some patients with MF [38]. Additional treatment options are available for patients with serum EPO ≥ 500 mU/mL. The erythroid maturation agent, luspatercept, has demonstrated anemia benefits in patients with MF and myelodysplastic syndrome/MPN with ring sideroblasts who carry the SF3B1 mutation [39, 40]. It is important to note that the studies that evaluated luspatercept in MF had small patient populations, and additional investigation is warranted to further evaluate safety and efficacy. Anabolic steroid medication such as danazol can also be used for the treatment of anemia in patients with MF [16, 25]. Danazol treatment has been associated with an anemia response in these patients, including those who are transfusion-dependent [41]. Immunomodulatory imide agents (IMiDs), such as thalidomide and lenalidomide, have also demonstrated an anemia benefit in patients with MF, including those who were transfusion-dependent [42, 43]. However, this benefit was not observed in patients with myeloid metaplasia with MF who received thalidomide [44] or those with MF treated with pomalidomide, another IMiD [45]. Importantly, various treatments can be combined with ongoing ruxolitinib treatment, although the coadministration of IMiDs with steroids is currently a topic of debate. The combination of ruxolitinib with prednisone, thalidomide, and danazol has been associated with an anemia benefit in patients with MF [46]. Similarly, luspatercept combined with ruxolitinib demonstrated transfusion independence in some patients with MF [39]. Details for studies of ruxolitinib in combination with other agents, including ongoing/exploratory trials, are shown in Table 1.

**Table 1:** Key clinical trials featuring ruxolitinib-based combination therapy in patients with MF and anemia

Agent (class) Study patients, *N*	Phase (NCT#)	Main inclusion criteria	Anemia results	Safety overview
Danazol [52] (androgen) *N* = 14	2 (NCT01732445)	• Anemia (Hb < 10 g/dL) • Age ≥ 18 years • ECOG PS ≤ 2 • ANC ≥ 1 × 10^9^/L • PLT, ≥ 50 × 10^9^/L	• 4/5 (80%) JAKi-naive patients had stable or increasing Hb • 5/9 (56%) patients who had received JAKi had stable or increasing Hb	**Hematologic grade ≥ 3 AEs**: 71% (*n* = 10) **Nonhematologic grade ≥ 3 AEs**: 14% (*n* = 2)
Luspatercept [39, 53] (TGFβ superfamily receptor ligand trap) *N* = 79	2 (NCT03194542)	• Anemia • Age ≥ 18 years • ECOG PS ≤ 2 • ANC ≥ 1 × 10^9^/L • PLT, ≥ 50 × 10^9^/L	• Mean Hb increase ≥ 1.5 g/dL from BL: NTD + RUX = 8/14 (57%); NTD, no RUX = 3/20 (15%) • RBC-TI ≥ 12 weeks during study: TD + RUX = 8/22 (36%); TD, no RUX = 4/21(19%) • ≥ 50% reduction in RBC transfusion burden: TD + RUX = 10/22 (46%); TD, no RUX = 8/21 (38%)	**TRAEs in ≥ 5% of patients**: • Hypertension, 13% • Bone pain, 9% • Diarrhea, 5% • 10% discontinued because of drug-related toxicity
Sotatercept [54] (TGFβ superfamily receptor ligand trap) Sotatercept monotherapy: *n* = 24 RUX combination cohort: *n* = 9	2 (NCT01712308)	• Anemia (Hb < 10 g/dL) • Age ≥ 18 years • Sporadic RBC transfusions, or TD	• ORR = TI + Hb increase ≥ 1.5 g/dL from BL for ≥ 12 consecutive weeks without RBC transfusion • 6/17 (35%) in sotatercept monotherapy cohort	**TRAEs**: • Grade 2 bilateral lower limb pain, *n* = 2 (1 patient in each cohort) • Hypertension, *n* = 1
		• **RUX combination cohort**: ≥ 6 months RUX with stable dose for ≥ 2 months	• **RUX combination cohort**: ORR in 1/8 (12.5%)	
Thalidomide [55] (immunomodulatory agent) *N* = 23 (*n* = 15 evaluated)	2 (NCT03069326)	• Age ≥ 18 years • ECOG PS ≤ 2 • ANC ≥ 1 × 10^9^/L • PLT, ≥ 50 × 10^9^/L • Suboptimal response, or refractory to RUX single-agent therapy • RUX treatment for ≥ 3 months, and stable dose for ≥ 4 weeks before enrollment	**Cycle 3**: • Trend toward increase in Hb over time	**Nonhematologic grade ≥ 3 AEs:** • Limb edema, diverticulitis, hypertension, syncope (*n* = 1 each) • Thromboembolic event and grade 3 neutropenia, *n* = 1
Prednisone, thalidomide, and danazol [46] *N* = 72 (*n* = 53 in combination therapy group)	Pilot study (ChiCTR1900025219)	• Age ≥ 18 years • SV ≥ 450 cm^3^ • Peripheral blood blasts < 10% • ECOG PS ≤ 2 • DIPSS: int-1, int-2, or high-risk • ANC ≥ 1 × 10^9^/L • PLT, ≥ 50 × 10^9^/L	**Combo therapy group vs RUX monotherapy:** • Anemia response: 56% vs 0% • Hb increase ≥ 10 g/L: 66% vs 0% • Hb increase ≥ 20 g/L: 38% vs 0%	**Combo therapy group vs RUX monotherapy:** • New or worsening anemia: 21% vs 58% • No grade 3/4 nonhematologic AEs occurred
Erythropoiesis-stimulating agents [38] *N* = 59	Retrospective study (completed)	• Anemia (Hb < 10 g/dL) • IPSS: int-2 or high (int-1 for some patients in compassionate use)	• Anemia response: 54% • Median time to anemia response: 4 mo	• Mild nausea, no other thrombotic events or toxicities reported

*AE*, adverse event; *ANC*, absolute neutrophil count; *bid*, twice daily; *BL*, baseline; *DIPSS*, Dynamic International Prognostic Scoring System; *ECOG PS*, Eastern Cooperative Oncology Group Performance Status; *Hb*, hemoglobin; *int*, intermediate; *MF*, myelofibrosis; *NTD*, non-transfusion dependence; *ORR*, overall response rate; *PLT*, platelet count; *PV*, polycythemia vera; *qd*, once daily; *qw*, once weekly; *RBC*, red blood cell; *RBC-TI*, red blood cell transfusion independence; *RUX*, ruxolitinib; SV, spleen volume; *TD*, transfusion dependence; *TI*, transfusion independence; *tid*, 3 times daily; *TRAE*, treatment-related adverse event

### MF treatment considerations in patients with MF-related anemia

In general, MF treatment is initiated as early as possible for symptomatic patients in my practice, as supported by clinical trial evidence. A pooled analysis of the COMFORT I/II trials suggested that earlier ruxolitinib initiation in patients with intermediate-2 or high-risk MF was associated with improved clinical outcomes including fewer anemia events [47]. In addition, a post hoc analysis of the phase 3 JUMP trial demonstrated that a lower IPSS score at treatment initiation was associated with better spleen response rates, suggesting that ruxolitinib treatment earlier in the disease course improves response [48]. This should be balanced by possible lead-time bias and the known relationship between lower MF disease stage and better spleen response in patients treated with ruxolitinib [49]. Nonetheless, treating MF as early as possible, before the onset of MF-related anemia, should improve outcomes, both because of the direct benefit of early intervention and indirectly due to potentially avoiding the negative outcomes associated with MF-related anemia itself.

**Fig. 2:**
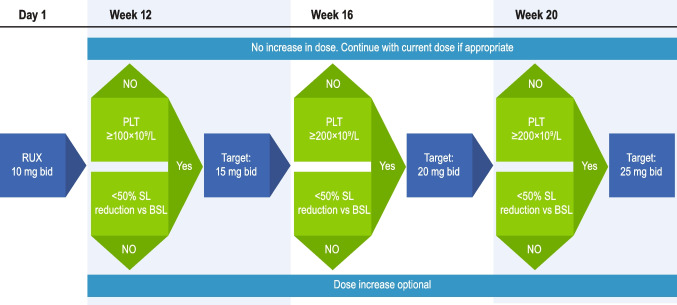
REALISE dosing strategy for ruxolitinib in patients with MF-associated anemia. bid, twice daily; BSL, baseline spleen length; PLT, platelet count; RUX, ruxolitinib; SL, spleen length. Figure reproduced from Cervantes F, et al. *Leukemia*. 2021;35(12):3455–3465, under the terms of the Creative Commons Attribution 4.0 International (CC BY 4.0) license (https://creativecommons.org/licenses/by/4.0/). Edits for style were made

For patients who develop MF-related anemia, anemia is not a driver for primary treatment choice and therefore is managed based on my practice’s standard MF treatment algorithm (Fig. 1). In particular, ruxolitinib is not contraindicated in patients with anemia [17]. In the COMFORT I/II trials, ruxolitinib was associated with prolonged survival in patients with MF compared with controls, regardless of baseline anemia status [14]. Regarding the choice of ruxolitinib dose, my practice follows in many patients the approach evaluated in the phase 2 REALISE trial, which established a novel ruxolitinib dosing strategy for patients with anemia based on a lower ruxolitinib starting dose (10 mg twice daily [bid] with up-titration as necessary based on platelet counts and efficacy; Fig. 2) [12]. REALISE demonstrated that patients with baseline anemia experienced improvements in spleen size and MF-related symptoms with ruxolitinib treatment, and median Hb levels remained stable throughout the study, with red blood cell (RBC) transfusion requirements decreasing or remaining stable [12].

## Sample patient—Sect. 2

The patient was not interested in undergoing a hematopoietic stem cell transplant right at diagnosis and was prescribed ruxolitinib 15 mg bid, as recommended for platelet counts between 100 and 200 × 10^9^/L. During follow-up 3 weeks after treatment initiation, the patient reported feeling better, eating more, and tolerating the treatment well. Upon examination, the spleen was smaller in size, at 2 cm below the costal margin. Laboratory results showed Hb of 8.7 g/dL, a platelet count of 67 × 10^9^/L, and a WBC count of 14 × 10^9^/L. Due to the decrease observed in platelet count, the patient was now prescribed a decreased dose of ruxolitinib, at 10 mg bid.

After one more month of therapy, the patient reported feeling much better than before ruxolitinib. The spleen size remained at 2 cm below the costal margin, and laboratory reports showed Hb at 7.5 g/dL, a platelet count of 82 × 10^9^/L, and a WBC count of 17 × 10^9^/L.

### Treatment options for anemia resulting from JAK inhibition

Although MF itself can lead to anemia, JAK inhibition may also separately cause or exacerbate anemia, which often occurs early in treatment and gradually improves with long-term exposure [13, 14, 17–19, 50]. In the COMFORT studies, the number of patients with grade 3 or 4 anemia was higher for ruxolitinib compared with placebo; however, the lowest Hb levels were observed at weeks 8 to 12 of treatment and recovered to near-baseline levels by week 24 [14, 18, 19]. Furthermore, the number of patients with grade 3 or 4 anemia decreased over 42 months of treatment, with no patients reporting new or worsening grade 3 or 4 anemia after month 42 of treatment [13]. Importantly, new or worsening postbaseline anemia did not affect survival probability during ruxolitinib treatment in the COMFORT I/II pooled analysis [14]. In fact, patients with postbaseline anemia who received ruxolitinib had a survival advantage compared with the overall control group [14]. Likewise, transfusion dependence did not affect the survival benefit observed with ruxolitinib treatment in the COMFORT studies [20]. Similar to observations with ruxolitinib, in the JAKARTA studies of fedratinib in MF, a decrease in Hb levels was observed for 12 to 16 weeks, with a partial recovery observed afterward in the 400-mg group [50]. Taken together, these findings demonstrate that treatment-induced anemia as a result of JAK inhibition can be temporary.

**Fig. 3:**
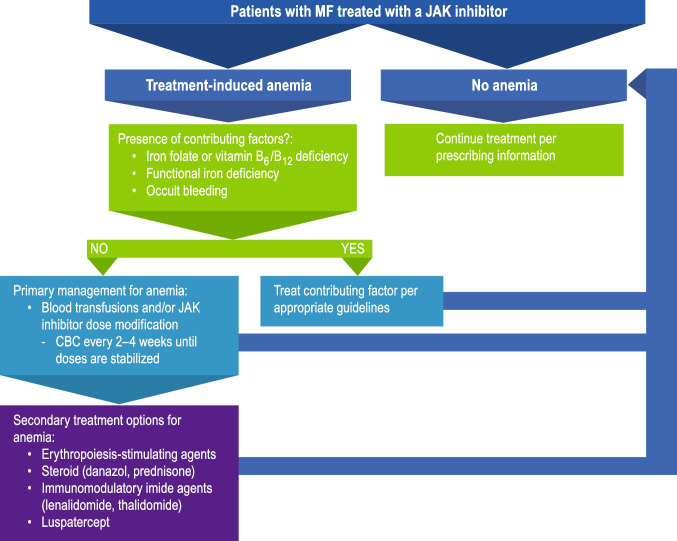
Treatment flow chart for patients with MF and treatment-induced anemia. CBC, complete blood count; JAK, Janus kinase; MF, myelofibrosis

In general, management for treatment-related anemia follows the same pattern described above for MF-related anemia, where contributing factors should first be assessed and treated appropriately. In the absence of non-MF-related contributing factors, primary management includes RBC transfusion and potential addition of secondary anemia treatments (Fig. 3). If transfusions and secondary treatment options are insufficient or burdensome, JAK inhibitor dose reduction can be considered to help improve anemia [17, 21]. After recovery of anemia to acceptable levels, ruxolitinib should be continued at the given dose or with subsequent modifications if necessary. Complete blood counts should be monitored every 2 to 4 weeks until doses are stabilized [16]. I try to avoid interruptions in therapy with ruxolitinib, as it has been reported that patients may have a significant rebound in symptoms within 7 to 10 days upon sudden interruption of ruxolitinib [16].

The JAK1/JAK2 inhibitor momelotinib, currently under investigation for patients with MF and anemia, has potent inhibitory activity against ACVR1/ALK2 and may become a second-line treatment option for patients who have to eventually stop ruxolitinib due to excessive anemia [30, 31]. This mechanism of action includes suppression of aberrant activation of hepcidin transcription in the liver and thus may improve iron homeostasis, facilitating normalized Hb levels and a decrease in transfusion requirements [30, 31]. In the phase 3 MOMENTUM trial of momelotinib versus danazol in patients with intermediate or high-risk MF previously treated with a JAK inhibitor, momelotinib provided superior clinical benefit as assessed by Myelofibrosis Symptom Assessment Form Total Symptom Score (MFSAF TSS) response and spleen response rate, as well as noninferiority for transfusion independence rate [51].

## Sample patient—Sect. 3

The patient continued ruxolitinib treatment and underwent a transfusion with packed RBCs. In addition, anemia medication was provided, including an ESA as serum EPO was < 500 mU/mL. Follow-up was scheduled for every 3 to 4 weeks. After 6 months of therapy, the patient’s Hb was 8.4 g/dL, the platelet count was 77 × 10^9^/L, and the WBC count was 12 × 10^9^/L. The spleen was no longer palpable, no transfusions were needed, and the patient reported no symptoms.

## Conclusions

Patients with MF endure burdensome symptoms and coexisting conditions as a result of their disease. In particular, patients commonly develop anemia, which can either be secondary to the disease or a result of MF treatment, further complicating disease management. Although MF treatment with JAK inhibitors can exacerbate anemia, evidence suggests that this is typically temporary and, as in the case of ruxolitinib, does not reduce survival contrary to MF-related anemia. MF treatment should be initiated as early as possible for symptomatic patients, ideally before the onset of MF-related anemia, to maximize clinical benefit. For those patients with MF who develop anemia, careful patient management, including RBC transfusions, secondary anemia treatments, JAK inhibitor dose modifications, and monitoring, can improve anemia to prevent further disease complications and improve clinical outcomes.
